# *Osteo-cise: Strong Bones for Life*: Protocol for a community-based randomised controlled trial of a multi-modal exercise and osteoporosis education program for older adults at risk of falls and fractures

**DOI:** 10.1186/1471-2474-13-78

**Published:** 2012-05-28

**Authors:** Jenny Gianoudis, Christine A Bailey, Kerrie M Sanders, Caryl A Nowson, Keith Hill, Peter R Ebeling, Robin M Daly

**Affiliations:** 1 NorthWest Academic Centre, Department of Medicine, University of Melbourne, Western Health, Melbourne, Australia; 2 Centre for Physical Activity and Nutrition Research, School of Exercise and Nutrition Sciences, Deakin University, 221 Burwood Highway, Burwood, Melbourne, 3125, Australia; 3 Allied Health, La Trobe University, Northern Health, Melbourne, Australia; 4 National Ageing Research Institute, Melbourne, Australia; 5 School of Physiotherapy, Curtin University, Perth, Australia

**Keywords:** Osteoporosis, High velocity resistance training, Falls prevention, Bone mineral density, Muscle function, Community program

## Abstract

**Background:**

Osteoporosis affects over 220 million people worldwide, and currently there is no ‘cure’ for the disease. Thus, there is a need to develop evidence-based, safe and acceptable prevention strategies at the population level that target multiple risk factors for fragility fractures to reduce the health and economic burden of the condition.

**Methods/design:**

The *Osteo-cise: Strong Bones for Life* study will investigate the effectiveness and feasibility of a multi-component targeted exercise, osteoporosis education/awareness and behavioural change program for improving bone health and muscle function and reducing falls risk in community-dwelling older adults at an increased risk of fracture. Men and women aged ≥60 years will participate in an 18-month randomised controlled trial comprising a 12-month structured and supervised community-based program and a 6-month ‘research to practise’ translational phase. Participants will be randomly assigned to either the *Osteo-cise* intervention or a self-management control group. The intervention will comprise a multi-modal exercise program incorporating high velocity progressive resistance training, moderate impact weight-bearing exercise and high challenging balance exercises performed three times weekly at local community-based fitness centres. A behavioural change program will be used to enhance exercise adoption and adherence to the program. Community-based osteoporosis education seminars will be conducted to improve participant knowledge and understanding of the risk factors and preventative measures for osteoporosis, falls and fractures. The primary outcomes measures, to be collected at baseline, 6, 12, and 18 months, will include DXA-derived hip and spine bone mineral density measurements and functional muscle power (timed stair-climb test). Secondary outcomes measures include: MRI-assessed distal femur and proximal tibia trabecular bone micro-architecture, lower limb and back maximal muscle strength, balance and function (four square step test, functional reach test, timed up-and-go test and 30-second sit-to-stand), falls incidence and health-related quality of life. Cost-effectiveness will also be assessed.

**Discussion:**

The findings from the *Osteo-cise: Strong Bones for Life* study will provide new information on the efficacy of a targeted multi-modal community-based exercise program incorporating high velocity resistance training, together with an osteoporosis education and behavioural change program for improving multiple risk factors for falls and fracture in older adults at risk of fragility fracture.

**Trial registration:**

Australian New Zealand Clinical Trials Registry reference ACTRN12609000100291

## Background

Osteoporosis and related fractures are a serious public health problem worldwide because of the associated morbidity, mortality and health care costs. In 2011, 1.2 million Australians were diagnosed with osteoporosis and another 5.4 million had osteopenia and are therefore at risk of fragility fracture [1]. Due to the demographic trend towards an ageing population, the incidence of osteoporosis-related conditions is projected to rise to three million people by 2021, with a fracture occurring every three and a half minutes unless effective prevention strategies are implemented [2]. For those that sustain a hip fracture, it is estimated that more than 20% will die within 6 to 12 months post fracture [3], almost 50% will require long-term nursing care [2] and up to 80% of those who survive will fail to regain their pre-fracture level of function [4].

Currently the medical paradigm of a ‘cure’ for osteoporosis is less than ideal because the pharmacological agents available are directed to those who have already had fragility fractures or have a high absolute fracture risk and the lowest bone density. However, in women it is estimated that more than 50% of fragility fractures occur in those with ‘osteopenia’ (BMD T-score −1.0 to −2.5 SD) and not ‘osteoporosis’ (T-score ≤ −2.5 SD) [5]. Furthermore, since most osteoporotic fractures are due to a fall or minimal trauma [6,7] there is considerable interest in identifying safe, effective and widely accessible community-based strategies for addressing multiple fracture-related risk factors, particularly reduced bone density, muscle wasting and weakness, poor balance and impaired gait and mobility which increase the risk of falling.

Current national and international consensus osteoporosis management guidelines recommend a combination of weight-bearing and resistance training with challenging balance exercises to improve multiple risk factors for falls and fracture [8]. At present however, there is no scientific consensus regarding the optimal mode and dose (type, frequency, duration and intensity) of exercise that can simultaneously enhance bone and muscle health and improve functional ability in older adults at risk of osteoporosis, falls and fractures. A meta-analysis of randomised controlled trials in older adults reported that multi-component exercise programs incorporating a combination of weight-bearing impact exercise (jogging, stair-climbing, jumping activities) and progressive resistance training (PRT) were most effective for maintaining bone mineral density (BMD) or preventing bone loss at clinically relevant sites such as the hip and spine [9].

Programs incorporating high intensity PRT alone are also particularly effective for improving muscle strength, mass and size [10] and may attenuate bone loss if they incorporate targeted hip and spine loading exercises [11,12]. However, the effectiveness of traditional PRT on other fall-related risk factors such as balance, gait, mobility and postural sway, are mixed [13-15]. This has been attributed in part to the fact that most PRT programs encourage slow velocity contractions (2 to 4 seconds concentric phase) at a moderate to high percentage of maximal force. However, many common tasks related to mobility and daily perturbations require rapid co-ordinated contractions within 50–200 msec, which is less than the time needed to achieve maximal muscle force (400–600 msec) [16]. Therefore, programs which focus on improving the ability to generate force quickly, which is often referred to as power training, and that are specific to tasks of daily living, are likely to be more relevant to improving muscle function and reducing the risk of falls.

The clinical relevance of maintaining lower extremity muscle power has been demonstrated in studies which have shown that relative to muscle strength, deficits in muscle power are a stronger predictor of disability and falls in older people [17,18]. Power training, which is also referred to as high velocity (HV) PRT, is characterised by rapid concentric (lifting or pushing) movements followed by a slower eccentric (lowering) phase. Several recent reviews and meta-analyses of the limited randomised controlled trials available have demonstrated that high velocity PRT is safe and feasible for older adults and may be a more effective training method for improving functional outcomes (e.g. chair rising time, stair climbing, walking speed) compared to traditional PRT, despite producing similar gains in muscle strength [19]. Moreover, there is also evidence from one previous intervention indicating that high velocity PRT is more effective for slowing the rate of bone loss than traditional PRT [20]. Based on these findings, we hypothesize that high velocity PRT may be the optimal training method for improving both bone health and muscle function. Whether the inclusion of high velocity PRT into an exercise program can reduce falls in the elderly has not been examined.

For older people, preventing falls is imperative as around 90% of all osteoporotic fractures, particularly hip fractures, occur as a result of a fall [21,22]. Fall-related injuries are also associated with considerable morbidity, mortality and healthcare utilization. It has been reported that approximately 30% of people who fall experience a serious injury, and 5 to 10% suffer a fracture with up to 1.5% suffering a hip fracture [23,24]. Falls are also associated with increased disability, loss of independence and reduced physical activity, which lead to reduced quality of life [25]. Regular exercise is known to be one of the most important strategies for reducing falls risk, but not all forms of exercise are equally effective. In a recent systematic review and meta-analysis of 54 randomised controlled trials (RCTs), exercise was found to reduce the risk of falls by 16% in older people [26]. In this study, it was reported that the most effective mode of exercise for preventing falls was high-challenge balance training and the inclusion of walking in any exercise program tended to attenuate the beneficial effects [26]. The authors’ earlier meta-analysis of RCTs reported that traditional moderate or high intensity PRT alone did not significantly reduce falls risk [15]. A more recent Cochrane review reported that multi-component group exercise, typically including resistance and balance training, reduced the rate of falls by 22% and falls risk by 17% in older adults aged 60 years and over [27]. In this review, exercise was the only intervention to reduce both fall rates and future falls risk; other interventions such as home modification strategies, vitamin D supplementation, medical review and cataract surgery only reduced one of these outcomes. While these findings support the concept that targeted exercise programs are likely to be most effective for optimising muscle function and reducing falls, from a public health perspective it is important to evaluate whether such programs can be successfully and safely translated from the research setting into the community.

One of the key challenges facing health-care professionals when implementing any community-based exercise programs is how to enhance adoption and adherence to the program and achieve sustained behaviour change. In older adults, there is considerable evidence that the use of behavioural theory-based strategies can effectively enhance the adoption to and maintenance of exercise programs [28,29]. Specifically, the Transtheoretical (Stages of Change) Model which matches an intervention to an individuals’ level of motivational readiness, and the Social Cognitive Theory (SCT) that includes both social and cognitive elements which can influence behaviour (e.g. self-efficacy, social support, self monitoring), appear to be particularly effective behavioural management strategies to maximise adoption, increase motivation and adherence, and minimise attrition from exercise programs [28,30,31]. There is also evidence that interventions which incorporate patient education can improve knowledge, sustainability in behaviour change and adherence to osteoporosis prevention guidelines [32,33]. Therefore, we believe that the design of any community-based osteoporosis, falls and fracture prevention program should include a targeted exercise program with soundly based behavioural strategies and patient education to achieve the greatest sustained benefits.

This paper describes the methods of the *Osteo-cise: Strong Bones for Life* trial; an 18-month community-based randomised controlled trial designed to evaluate the effectiveness and feasibility of an evidence-based, multi-modal exercise, osteoporosis education and behavioural change program for improving bone health, muscle power and function in community-dwelling older adults at increased risk for falls and fracture. Secondary aims of the study are to assess the effects of the intervention on: 1) trabecular bone microarchitecture; 2) lower limb and back muscle strength; 3) falls incidence, and 4) health-related quality of life. We will also conduct a cost-utility analysis to facilitate the cost effectiveness compared with other interventions targeted across a range of chronic disorders. The long-term goal is that the *Osteo-cise: Strong Bones for Life* program will serve as a model for future osteoporosis and falls prevention programs that can be implemented within the community with the aim of reducing fracture incidence and related risk factors for fracture, and consequently reduce osteoporosis-related health care costs and morbidity.

## Methods/design

### Study design

*Osteo-cise: Strong Bones for Life* is an 18-month multi-faceted randomised controlled trial involving community-dwelling men and women aged 60 years and over at increased risk of falls and fractures. The trial is divided into two phases: 1) a 12-month community-based structured and supervised intervention incorporating a multi-modal exercise program in conjunction with an osteoporosis education/awareness and a behavioural change program, and 2) a 6-month ‘*research to practise’* translational period in which each community-based health and fitness centre will aim to continue to implement the *Osteo-cise: Strong Bones for Life* program independently of research staff. This translational phase is designed to evaluate the sustainability of the program in the ‘real world’. The trial is managed by the Department of Medicine, Northwest Academic Centre, The University of Melbourne, Western Hospital, Australia and has been approved by the Melbourne Health Human Research Ethics Committee (HREC 2008.136). The study is also registered with the Australian New Zealand Clinical Trials Registry [Reference ACTRN12609000100291].

### Participants

Men and women aged 60 years and over living independently in the community in the Western suburbs and surrounding regions of Melbourne, Australia, who fulfil the defined inclusion criteria outlined below will be invited to participate in the study.

#### Inclusion/exclusion criteria

Eligibility to participate in the study will be based on a three step screening process. First, all participants who enquire about participating in the study will be initially screened via the telephone. Participants must meet the following criteria: i) aged ≥60 years; ii) BMI <40 kg/m^2^; iii) no history of osteoporosis; iv) not undertaken resistance training and/or weight-bearing impact exercise more than once a week in the past three months; v) be willing to be randomised into either the ‘*Osteo-cise’* intervention or usual care control group, and vi) be willing and able to travel to a local health and fitness centre three times per week for 18 months to complete the exercise program. Participants will be excluded from the study on the following criteria: i) current smoking; ii) current or prior (past 6 months) use of hormone replacement therapy (>0.625 mg/d premarin or equivalent estrogen); iii) having sustained a low trauma fragility fracture in the past 6 months; iv) any medical condition (e.g. type 1 diabetes, chronic kidney failure or liver disease, cancer) or use of medication known to influence bone metabolism or fracture risk (e.g. glucocorticoids, thiazide diuretics, anticonvulsants, bisphosphonates); v) initiating calcium or vitamin D supplementation in the preceding 6 months, or vi) expected travel of greater than 6 weeks in the next 18 months.

Potential participants will then have a DXA bone mineral density (BMD) scan and be eligible for randomisation if they are osteopenic (T-score between −1.0 and −2.5 SD) at the total hip, femoral neck or lumbar spine. Those with osteoporosis (T-score ≤ −2.5 SD) will be excluded and advised to consult their physician for follow-up care. Participants with normal BMD (greater than −1.0 SD) will complete a short falls and fracture risk questionnaire, adapted from Sanders et al. [34] (Table 1). Those with a total risk score of three points of higher will also be eligible to participate in the study. All eligible participants will be required to obtain approval from their local doctor to clear them of any contraindicated medical conditions to exercise, based on American College of Sports Medicine (ACSM) guidelines [8]. Written consent will also be obtained from all participants prior to commencing the program.

**Table 1:** **Falls and fracture risk questionnaire for inclusion into the *Osteo-cise: Strong Bones for Life *** **study**

**Risk Factor**	**Guidelines**	**Value**	**Score**
History of Falling ^1^	Self-reported risk of falling (2 or more falls past year)	3	
Low trauma fracture ^2^	Since age of 50 years	3	
Parental hip fracture	Did either of your parents suffer a hip fracture?	3	
Age	>75 years	2	
Menopause status	Early menopause or hysterectomy (<45 yr)	2	
Inability to rise from a chair without using arms	Are you able to rise from a chair without using your arms?	2	
Medication use	Are you currently taking four or more medications?	2	
Below average BMD	Proximal femur bone density T-score between 0.0 to –1.0 SD	2	
Thinness	BMI <20	2	
High risk of low vitamin “D” or calcium	“Do you spend less than 10 minutes per day outdoors (with part of your body exposed to sunlight), without taking vitamin D supplements”		
	OR	1	
	“Do you avoid, or are you allergic to milk or dairy products, without taking calcium supplements”		
**Score ≥ 3 = Eligible for the study**		**Total**	

^1^ A fall is defined as an event which results in a person coming to rest inadvertently on the ground or floor or other lower level.

^2^ A low trauma fracture is defined as any fragility fracture (e.g. hip, spine, forearm, humerus, pelvis, ribs) from a standing height or less.

#### Recruitment of participants

Participants will be recruited into one of two cohorts approximately 3 months apart in order to assist with the timely implementation and management of the intervention. A number of different strategies will be used to attract potential participants based on our experience from conducting previous trials in older adults [35,36]. This will include developing advertisements (paid), media releases and general interest articles for a range of media including local radio, television, state and local newspapers and council/community newsletters. Flyers will also be distributed to health professionals and community groups within the local area and posters displayed on notice boards at Rotary and Probus clubs, lawn bowling clubs, senior citizens groups, hospitals, medical centres, supermarkets and pharmacies. Information sessions will be delivered to local community seniors groups. Finally, participants already enrolled in the study will also be asked to recommend the study to family and friends.

#### Randomisation

**Figure 1:**
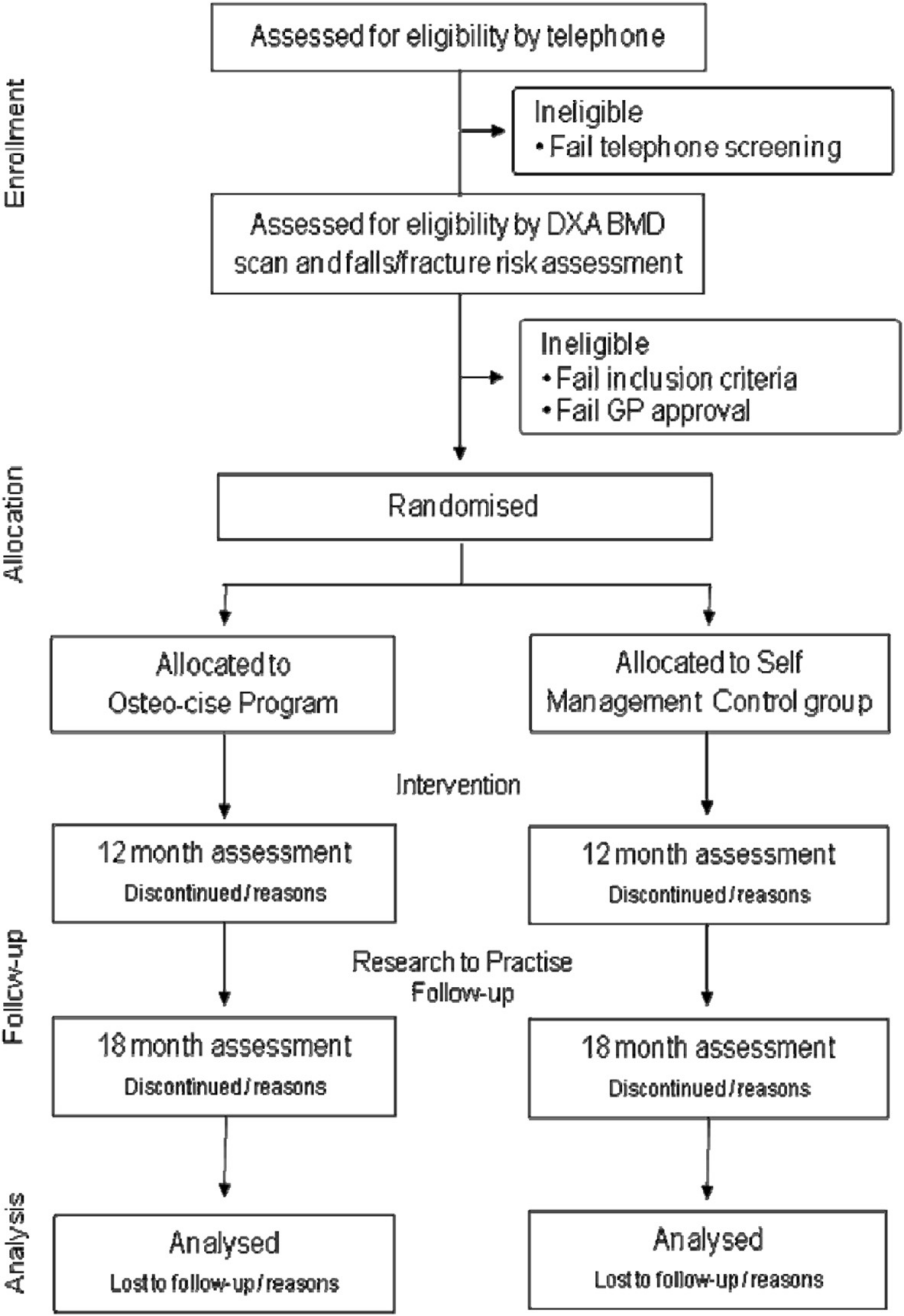
Flow diagram of the progress from screening to the final follow-up assessment.

Following baseline testing participants will be randomised into either the *Osteo-cise: Strong Bones for Life* program or a self-management control group, stratified by sex, by an independent staff member not involved in the study using a computer generated random numbers table (Microsoft Excel). To avoid any potential contamination any couples enrolled in the study will be randomised into the same group. A flow diagram of the study protocol is outlined in Figure 1.

### Intervention

#### Osteo-cise: Strong bones for life program

*Osteo-cise: Strong Bones for Life* is a community-based program that includes four key components: 1) Osteo*-cise*: a multi-modal targeted osteoporosis and falls prevention exercise program; 2) Osteo*-Adopt*: a behavioural change program designed to optimise adherence to the Osteo-*cise* program; 3) Osteo*-Ed*: an osteoporosis awareness and education program for the study participants, and 4) Osteo*-Instruct*: a train-the-trainers workshop designed to update the exercise trainers with the latest osteoporosis prevention and management information and to instruct them on the aims and structure of the program. The structured exercise program is designed to run over 12 months and to be conducted within local community leisure or recreational health facilities under the supervision of *Osteo-cise* trained exercise instructors. Managers of local health and fitness centres within the western suburbs of Melbourne will be contacted by phone and invited to participate in the study. All interested managers and relevant staff will be given a detailed presentation outlining the program and requirements. Those that agree will then be asked to recommend suitably qualified exercise trainers to undertake the Osteo-*Instruct* ‘train-the-trainers’ workshop to implement the exercise program at their facility. Specific details on the program’s four key components are provided below.

##### *1. ‘*Osteo*-cise’: Targeted Osteoporosis Exercise Program*

Participants randomised to the Osteo*-cise* group will be instructed to train at their nearest *Osteo-cise* health and fitness centre on three non-consecutive days per week for 18 months. The gymnasium membership for all participants during the initial 12 months will be subsidised. Exercise sessions will be conducted in small groups (a maximum of 10 per group) and monitored by qualified and certified exercise trainers who have completed the Osteo-*Instruct* training workshop. Before commencing the program, all participants will be required to attend an orientation session at their selected leisure facility to become familiar with the aims and design of the training program and the rules and regulations of each health and fitness centre.

The initial 12 months of the *Osteo-cise* program will be divided into a 4-week ‘Adoption Phase’ followed by four distinct 12-weekly mesocycles (Table 2). Each mesocycle is further divided into three 4-weekly microcyles which are designed to promote a sense of achievement and become progressively more challenging. Every session of the 4-week ‘Adoption Phase’ and the first week of each mesocycle will be supervised by *Osteo-cise* trainers. For the remaining 11 weeks of each mesocycle, participants will attend one scheduled supervised session per week with their designated trainer and two additional sessions independently or with fellow participants. All participants will be instructed to consult other on-site gym staff if they require assistance with their exercise program. Each session is intended to take approximately 60 minutes to complete and will comprise of a warm-up, high velocity progressive resistance training (HV-PRT) using machine and free weights, moderate impact weight-bearing exercises, high challenge balance and mobility exercises, core muscle stabilization exercises and flexibility (stretching) exercises. All programs will be individually tailored and designed to follow the key training principles of specificity and progressive overload. To assist trainers with exercise selection for each participant and to ensure correct technique and exercise progression, trainers will be provided with an *Osteo-cise* exercise manual which will include detailed explanations and illustrations for all key exercises.

**Table 2:** **Structure of the *Osteo-cise: Strong Bones for Life *** **exercise program and training doses for 12-months**

**Mesocycle**		**Microcycle**	**Intensity**	**High-Velocity PRT**				**Impact Exercises**			**Functional Training**		
**Phase**	**Week**	**Training Phase**	**RPE**^**1**^	**Sets**	**Reps**	**Speed**^**2**^	**Rest**	**Sets**	**Reps**	**Rating**^**3**^	**Sets**	**Reps**	**Progression**
#1	1-2	Orientation to	3 - 4	2	12-15	Slow	1-2	3	10-20	Low	2		Fit Ball and
	3-4	Training	3 - 4	2	12-15	Slow	1-2	3	10-20		2		Standing Balance
#2	5-8	Preparatory #1	3 - 4	2	10-15	Slow	1-2	3	10-20	Low	2		Fit Ball and
	9-12	Challenge #1	5 – 8	2	8-12	Rapid	2	3	10-20	to	2		Standing Balance
	13-16	Consolidation #1	5 – 8	2	8-12	Rapid	2	3	10-20	Moderate	2	All	
#3	17-20	Preparatory #2	3 - 4	2	10-15	Slow	1-2	3	10-20	Moderate	2	functional	Standing Balance
	21-24	Challenge #2	5 – 8	2	8-12	Rapid	2	3	10-20		2	training	and Dynamic
	25-28	Consolidation #2	5 – 8	2	8-12	Rapid	2	3	10-20		2	30-60	Functional
#4	29-32	Preparatory #3	3 - 4	2	10-15	Slow	1-2	3	10-20	Moderate	2	seconds or	Standing Balance
	33-36	Challenge #3	5 – 8	2	8-12	Rapid	2	3	10-20	to	2	until fatigue	and Dynamic
	37-40	Consolidation #3	5 – 8	2	8-12	Rapid	2	3	10-20	High	2		Functional
#5	41-44	Preparatory #4	3 - 4	2	10-15	Slow	1-2	3	10-20	High	2		Standing Balance
	45-48	Challenge #4	5 – 8	2	8-12	Rapid	2	3	10-20		2		and Dynamic
	49-52	Consolidation #4	5 – 8	2	8-12	Rapid	2	3	10-20		2		Functional

^1^ RPE = Rating of Perceived Exertion; ^2^ Speed: slow speed = traditional resistance training (2–3 sec concentric and 2–3 sec eccentric contractions); rapid speed = high velocity training (rapid concentric and 2–3 sec eccentric contractions); ^3^Impact rating: Low = Ground Reaction Force (GRF) ~ 1–3 x body weight, Moderate = GRF ~ 3–6 x body weight, High = GRF ~ 6–9 x body weight: Rest in minute.

a) High velocity progressive resistance training (HV-PRT) or power training

HV-PRT is a type of resistance training whereby the concentric (lifting) phase of the exercise is performed as rapidly as possible in order to maximise both movement speed and muscle force. A combination of pin-loaded machine weights, pulleys and free weights will be used to overload the specific muscle groups that attach to skeletal sites prone to osteoporotic fracture, particularly the hip and spine. Trainers will be advised to choose from the following core exercises: squats, leg press, lunges, bench step ups, hip extension, hip flexion, hip abduction, hip adduction, calf raises, back extension, lateral side bends, latissimus dorsi pull down (or seated row) and a combination of abdominal and core stability exercises. Where possible, participants will be asked to perform the exercises whilst standing in an attempt to optimise balance and muscle function. Additional exercises including bench press, military press, bicep curls, tricep extension and shoulder, lumbo-pelvic and spine stabilisation exercises will also be rotated throughout the program to ensure the development of muscle balance. For each exercise card a total of six resistance exercises will be performed in each training session, with new exercises introduced into the program at the beginning of each new mesocycle (i.e. every 12 weeks) where appropriate.

Table 2 outlines the recommended training intensity and the number of sets and repetitions to be completed during each training phase of the 12-month structured program. Training during the first 4 weeks (Adoption phase) will be at low intensity and low velocity to allow the participants to master the correct lifting techniques and to become familiar with the training principles. Thereafter, the emphasis of the program will shift to HV-PRT for the lower extremity exercises. Exercise intensity will be monitored by the trainers using the Rating of Perceived Exertion scale (RPE), or the Rating of Perceived Difficulty for balance exercises, on the modified BORG 1 to 10 RPE scale. Participants will be expected to exercise at a level that corresponds to an RPE of between 5 and 8, which represents hard to very hard exertion.

b) Moderate impact weight-bearing exercise

The weight-bearing impact exercise component of the program will consist of a series of lower limb exercises aimed at specifically loading the hip. We have developed a battery of 27 bone friendly exercises which were found to be safe and effective for improving femoral neck and lumbar spine BMD in middle aged and elderly men [36,37]. Trainers will select appropriate weight-bearing exercises from the *Osteo-cise* manual based on each participant’s ability and/or on the existence of any previous medical conditions (e.g. knee osteoarthritis). Exercises are divided into three categories: Level 1 - Stationary movements (e.g. stomping, mini tuck jumps); Level 2 - Forward/Backward movements (e.g. box step-ups, backward and forward pogo jumps); and Level 3 - Lateral/Multidirectional movements (e.g. side-to-side shuffle, lateral box jumps). Each category has three levels (Easy, Moderate, Hard) based on the degree of difficulty and the magnitude of the impact forces (ground reaction forces) associated with each exercise [36]. A total of two to three weight-bearing impact exercises will be included in each training session. For each exercise, participants will perform three sets of 10 to 20 repetitions per session, equating to a total of 60 to 180 impacts per session. This dose was shown to be effective for increasing hip and spine BMD in our previous intervention in older men [36]. Training intensity will be progressively increased by increasing the height of jumps and/or by adding additional weight (i.e. holding dumbbells, weighted vest) or introducing multidirectional movement patterns.

c) Balance and mobility training

The balance training component will incorporate a variety of static and dynamic balance exercises aimed at improving posture and anticipatory/reactive balance and mobility. Exercises will fall into three categories: 1) Fit-ball exercises (e.g. fit-ball sitting with heel lifts), 2) Standing balance exercises (e.g. single leg standing), and 3) Dynamic functional exercises (e.g. heel-toe walking). Two challenging balance exercises will be performed in each session, with each exercise held for up to 30 seconds (e.g. 30 seconds of single leg standing) or performed for a given number of repetitions (e.g. 10 to 20 lateral weight shifts whilst sitting on a fit-ball). Once an exercise is no longer challenging for the participant they will be instructed to progress to the next level of difficulty by changing the exercise using the following progressions: closing eyes, reducing base of support, confounding visual fixation (e.g. head turns), changing centre of mass (e.g. arm/leg lifts) or adding a secondary manual or cognitive task. The type of balance exercises and starting level of difficulty will be prescribed by the trainer according to each participant’s functional ability.

d) Core and hip muscle stabilisation exercises

The key muscle groups targeted in this component of the exercise program include the rotator cuff (supraspinatus, infraspinatus, teres minor and subscapularis), scapular stabilisers (middle and lower trapezius, rhomboid major and minor, and serratus anterior), transverse abdominis and pelvic floor muscles. These muscles help to maintain good posture and trunk stability, which are important for balance and maximising muscle power [38]. Participants will complete two stabilising exercises per session using light to moderate resistance. For the rotator cuff and scapular stabilizers exercises, participants will perform two sets of 10 to 15 repetitions and up to 50 repetitions for the abdominals.

##### *2. ‘Osteo-Adopt’: Implementation of behavioural modification strategies*

An important component of this study is to develop a holistic community-based program that will provide older adults with the skills required to manage their own musculoskeletal health and promote behaviours that encourage adoption and maintenance of lifelong exercise participation. Increasing each participant’s motivation and readiness to exercise is crucial for long-term maintenance of musculoskeletal health. Consequently, widely established behavioural principles, particularly the Transtheoretical (Stages of Change) Model (TTM) and Social Cognitive Theory, will be utilised to initiate exercise adoption and guide long-term maintenance [30,31]. Fundamental to the TTM model are a number of cognitive and behavioural strategies that help individuals progress from lower to higher levels of motivational readiness, with the aim of modifying attitudes and fostering specific behaviours. Several approaches for improving adoption and adherence to exercise programs will be applied, including social support for physical activity, positive and social reinforcement, overcoming bar1riers to behaviour change, goal setting and self-monitoring.

Social support will be enhanced by organising participants into small exercise groups that facilitate socialisation and interaction. The development of relationships with other participants in the *Osteo-cise* program (‘buddy’ system) and telephone calls from the research staff or exercise trainers will also be used to optimise enjoyment of the program and hence adherence, as will quarterly newsletters aimed at providing information of interest (e.g. study updates, upcoming events and relevant scientific findings). In addition, at 3-monthly intervals trainers will ask participants to complete a Behavioural Evaluation Form which requires the establishment of short- and long-term exercise goals and helps to recognise motivating factors and potential barriers to attaining these goals. Strategies to overcome these barriers may then be identified and discussed by participants together with their trainer. Trainers will also endeavour to prevent lapses by calling participants who have missed three consecutive training sessions and discuss strategies to improve adherence. Finally, positive reinforcement will be achieved through self-monitoring of progression marked by the completion of the exercise cards.

##### *3. ‘Osteo-Ed’: Osteoporosis education and awareness program*

Participants will be invited to attend three osteoporosis-related community educational seminars presented by the research staff. These sessions will aim to improve knowledge and understanding of risk factors and preventative measures for osteoporosis, falls and fractures so that participants are encouraged to actively manage their health. The content of Osteo*-Ed* will be adapted from educational resources developed by Osteoporosis Australia that are based on current scientific evidence. Topics covered will include: 1) Osteoporosis – What are the risk factors?; 2) Exercise for optimal bone and muscle health; and 3) Nutrition for healthy bones. The Osteo*-Ed* seminars are intended to be informal sessions whereby questions and open discussion are encouraged. Each will run for approximately 60 minutes and will take place at selected *Osteo-cise* community leisure centres.

##### *4. ‘Osteo-Instruct’: Exercise instructors training workshop*

All exercise trainers employed to work on the *Osteo-cise: Strong Bones for Life* program will be required to attend a one-day training workshop that will cover information about the latest research on osteoporosis and falls; current treatment and management options; challenges facing older adults with or at risk of osteoporosis; basic nutrition for healthy bones; the benefits of exercise for preventing and managing osteoporosis and falls, including the most appropriate type, mode, intensity and frequency of training; safe exercise progressions; contraindicated exercises; and behavioural management strategies to enhance compliance to exercise. In addition, the Osteo*-Instruct* workshop will teach trainers how to identify early cues to poor adherence and strategies to prevent lapses and promote individuals’ self-confidence in their ability to undertake the exercise program. Importantly, the workshop will also familiarise the trainers with the design and specific aims of the *Osteo-cise* program. All trainers will be provided with a copy of the *Osteo-cise* training manual, which will contain all the essential information that is needed to implement the training program. This includes the rationale behind the program, training principles for optimal bone health, and a detailed description of each program component including progressions of exercise volume and intensity as well as illustrations of recommended exercises. Safety considerations, such as injury prevention and exercises to avoid for various disease states, will also be clearly highlighted in the trainer’s manual.

Trainers will be provided with a folder containing each study participant’s personal details, including relevant medical history and a copy of their physician's approval to exercise. Trainers will be required to document any contact made with participants throughout the intervention in this folder as well as any injuries or adverse events that occur as a result of the exercise sessions. The research team will maintain regular contact with the trainers and visit all health and fitness centres during each training cycle to ensure that the trainers are up-to-date with the program and that the quality of the program is being upheld and delivered as intended.

### Self-management control group

The self-management control group will serve as the comparison group in the intervention. Participants assigned to this group will receive general consumer information taken from Osteoporosis Australia fact sheets, including: “What is osteoporosis?”, “What are the risk factors for osteoporosis?” and “Calcium, vitamin D and exercise tips for preventing osteoporosis”. The aim is to enable these participants to actively take charge of their own musculoskeletal health and make educated decisions to seek further medical information and appropriate services to help prevent osteoporosis, falls and fractures. This group will undertake exactly the same testing protocol as the intervention group, but will not participate in the structured *Osteo-cise* program.

### Calcium and vitamin D supplementation

To ensure that daily calcium intakes meet the current recommended dietary intake of 1000 to 1300 mg/d for men and women aged over 50 years and that vitamin D levels are close to optimal (at least 60 to 75 nmol/L), participants in both groups will be requested to consume 2x350 mg elemental calcium tablets (Blackmores Total Calcium) and a single 1000 IU vitamin D_3_ capsule (Blackmores Vitamin D_3_) each day. All supplements will be provided free of charge.

### Follow-up period

During the final 6-month *‘research-to-practise’* phase, participants in the *Osteo-cise* program will be invited to continue the training program which will be administered by staff at each health and fitness centre with little or no input from the research staff. This translational phase is designed to evaluate the sustainability of the program in the ‘real world’. Participants in both groups will continue to take vitamin D and calcium supplementation over this period dispensed at no personal cost.

### Sample size

In order to calculate the number of participants required for this study, power calculations were performed based on BMD and functional muscle power results from previous exercise intervention trials [37,39]. It was estimated that 60 participants in each group would provide at least 90% power to detect a 1.8% difference (assuming a standard deviation [SD] of 3.5) for the change in femoral neck BMD at p < 0.05 (two-tailed). For functional muscle power, we estimated that 24 participants would provide 90% power to detect a 15% difference for the change between the groups (two-tailed, P < 0.05) assuming a SD of 15%. Therefore with an anticipated 20% dropout rate, a minimum of 72 participants per group will be required (144 in total).

### Outcome measures

A summary of the outcome measures to be collected is shown in Table 3. Participants will attend four clinic visits throughout the study (baseline, 6, 12 and 18 months). All clinical testing will take place in the Department of Medicine at the University of Melbourne, Western Hospital, Footscray, with the exception of the blood collection and laboratory testing for routine biochemistry and hormonal measures which will be completed by an external pathology laboratory.

**Table 3:** **Summary of the measures to be collected for the *Osteo-cise: Strong Bones for Life *** **study**

**Variables**	**Data Collection Method**	**Data Collection Points**			
		**Baseline**	**6 months**	**12 months**	**18 months**
**Primary outcome measures**					
Hip and spine BMD	DXA hip and spine scan	x		x	x
Functional muscle power	Stair climbing test	x	x	x	x
**Secondary outcome measures**					
Muscle power	Leg press (3-RM)	x	x	x	x
	30 second sit-to-stand	x	x	x	x
Muscle strength	Leg press and lat pulldown (3-RM)	x	x	x	x
Muscle function and balance	Functional reach test	x	x	x	x
	Four-square step test	x	x	x	x
	Timed-up-and-go (TUG)	x	x	x	x
Anthropometry and body composition	Height and weight	x	x	x	x
	DXA lean mass and fat mass	x		x	x
Trabecular bone microarchitecture	MRI knee	x			x
Falls	Monthly falls calendars	Monthly calendar			
Adverse events	Monthly falls calendars	Monthly calendar			
Cost effectiveness	EuroQol (EQ-5D)	x	x	x	x
Quality of life	SF36 questionnaire	x	x	x	x
**Other measures**					
Diet	24-h food recall questionnaire	x	x	x	x
Lifestyle and medical history	Questionnaire	x	x	x	x
Biochemistry and hormonal	Serum 25-hydroxyvitaminD				
		x	x	x	x
	Parathyroid hormone (PTH)	x		x	x
	Routine biochemistry	x		x	x
Physical activity	CHAMPS questionnaire	x	x	x	x
Historical physical activity	Questionnaire	x			
Osteoporosis health beliefs and knowledge	Osteoporosis Knowledge Test (OKT)	x		x	x
Osteoporosis attitudes and prevention behaviours	Osteoporosis Health Belief Scale (OHBS)	x		x	x

#### Primary outcomes

The primary outcome measures will be changes in proximal femur and lumbar spine BMD and lower limb functional muscle power.

##### *Bone mineral density (BMD)*

Areal bone mineral density (aBMD, g/cm^2^) will be measured at the lumbar spine (L1-L4) and proximal femur (total hip and femoral neck) using a Hologic Discovery W dual-energy X-ray absorptiometry (DXA) machine with the APEX Software v3.2 (Hologic, Bedford, MA). Standardized procedures for participant positioning and scan analysis will be used for all scans [40]. All follow-up measurements will be analyzed using the ‘comparison’ feature of the Hologic APEX 3.2 analysis program. The short term co-efficient of variation (CV) for repeated measurements of aBMD in our laboratory is 0.8% for lumbar spine, 0.8% for total hip and 1.5% for femoral neck aBMD.

##### *Functional muscle power*

Functional muscle power will be assessed by the Timed Stair Climb Test [41]. Participants will be instructed to climb a flight of stairs (10 steps, 14 cm rise per step) as quickly as possible without using the handrails or any other aid. Stair climbing power will be calculated according to the method described by Lazowski et al. [42].

(1) $$\text{SCP (W) = }\frac{\text{body weight }\left(\text{kg}\right)\text{ x gravity }\left({\text{ms}}^{-1}\right)\text{ x step height }\left(\text{m}\right)\text{ x no}.\text{ of steps}}{time(s)}$$

The short term co-efficient of variation for repeated measurements of this test in our laboratory is 2.2%.

#### Secondary outcome measures

The secondary outcome measures will include: changes in lower limb and back maximal muscle strength, power, balance and function, MRI-assessed distal femur and proximal tibia trabecular bone micro-architecture, body composition, falls incidence and health-related quality of life.

##### *Muscle strength and power*

Maximum muscle strength of the lower limbs and back will be estimated by three repetition maximum (3-RM) testing on a LC20 weights machine (GPI Bodyworx, Gym Co, Yarraville, VIC). A 3-RM test is the maximum weight that can be lifted for three complete repetitions of an exercise whilst maintaining good technique. The bilateral leg press exercise will be employed to determine maximal leg strength (knee extensors) and the seated row will be used to determine maximal back strength. The following formula used by Wathen et al. [43] will be used to predict each participant’s 1-RM, which represents the maximum weight that can be lifted for one complete repetition of an exercise:

(2) $$1\text{RM }=\;100\text{ x }\left[3\text{RM load}\right]/(48.8\;+\;53.{8\text{ xe}}^{(-}{}^{0.075\text{x}3)})$$

Peak muscle power of the lower limbs will be recorded on the bilateral leg press machine at five different loads (40%, 50%, 60%, 70% and 80% of the calculated 1-RM) by using the *Gymaware* power monitoring system (Kinetic Performance Technology, Canberra). Peak muscle power at each of the five loads will be assessed following the 3-RM testing and the calculation of 1-RM. All participants will complete two repetitions in succession at each 40%, 50%, 60%, 70% and 80% of their calculated 1-RM, with a 60 second rest between each test. Participants will be instructed to perform the concentric phase of the exercise as rapidly as possible but the eccentric (lowering) phase at a normal, controlled speed. For each test load, the highest peak power from the two repetitions and the corresponding velocity will be recorded.

##### *Muscle function and performance*

A series of commonly used functional tests will be used to assess lower limb function, dynamic balance and gait.

The *four square step test (FSST)* provides a measure of dynamic standing balance and stepping speed in four different directions [44]. To complete this test, participants will be required to step forward, sideways, and backwards over four canes resting flat on the floor in a cross formation, moving first in a clockwise direction and then counter-clockwise to return to the starting position. This test has been shown to have high interrater (ICC = .99) and retest reliability (ICC = .98) [44]. With a cut-off score of more than 15 seconds, the test has an 89% multiple faller sensitivity and 85% specificity for non-multiple fallers [44]. The test also has a positive predictive value of 86% for detecting a history of falls among community-living adults [44].

The *timed-up-and-go test (TUG)* is a measure of dynamic balance during three commonly performed functional activities: standing up and sitting down in a chair, walking and turning [45]. To increase the level of difficulty, participants will be asked to perform this test whilst simultaneously completing the cognitive task of counting backwards from 100 in 3's. This test has an established interrater reliability of ICC = .99 and prediction rate of 87% for identifying fallers and non-fallers when performing the test with an added cognitive task [46].

The *30-second sit-to-stand test* provides a measure of lower-extremity muscle strength where participants will be instructed to stand fully upright and then return to the seated position as many times as possible in 30 seconds [47]. This test has been shown to have good reliability with a test-retest intraclass correlation of 0.84 to 0.92 in a community-dwelling sample of older men and women aged 60 years and over [47].

The *functional reach test (FRT)* will be used to assess bilateral stance dynamic balance [48]. In this test, participants stand next to a wall unsupported and are asked to reach as far forward as possible without overbalancing. This test has been shown to be a valid tool with good interrater reliability (ICC = 0.98) and retest reliability (ICC = 0.92) [48] and has been shown to discriminate between healthy older people and Parkinson’s disease patients who were fallers and non-fallers [49,50].

##### *Trabecular bone micro-architecture*

All participants will be invited to have a Magnetic Resonance Imaging (MRI) scan of the left knee at baseline and 18 months to assess the effects of the intervention on trabecular bone micro-architecture at the distal femur and proximal tibia. MRI of the left knee will be acquired on a 3.0 T MR scanner (General Electric, Milwaukee, WI) using an eight-channel phased array knee coil (General Electric Medical Systems, WI). Images will be acquired with an axial 3D, phase cycled, fully balanced, steady-state coherent imaging pulse sequence (3D FIESTA-C), utilising the following parameters: repetition time ~11.8 ms, echo time 1.0 ms, flip angle 60 degrees, resolution 0.195 × 0.195, 512 × 384 matrix, slice thickness 1 mm and a scanning time ~16 minutes. A total of 50 slices will be acquired with a field of view of 10 cm.

Analysis of trabecular bone micro-architecture outcomes will be performed using an interface description language (IDL)-based image software program (RSI, Boulder, CO). Image processing consists of five steps including coil sensitivity correction, trabecular region segmentation, manual region adjustment, registration and bone/marrow thresholding, as described in detail by Newitt et al. [51]. The bone/marrow binary images will be generated by calculating a global threshold based on a dual reference limit and assuming a biphasic model [52]. These processed images will then be analysed for apparent structural parameters slice by slice (in 2D), similar to a previously described processing method [53]. For the distal femur, a 30 mm (30 slice) region of interest (ROI) will be analysed starting 5 mm from the most distal point of the femur. For the proximal tibia, a 15 mm (15 slice) ROI will be analysed starting 5 mm from the bone’s most proximal point. Within each axial image (or slice), the ROI will be selected consisting only of trabecular bone and marrow, with a border approximately 2 mm from the inner edge of the cortical bone boundary. Outcome variables after analysis will include: apparent bone-volume over total-volume fraction (BV/TV), apparent trabecular separation (TbSp), apparent trabecular plate thickness (TbTh) and apparent trabecular plate number (TbN) calculated for each slice.

##### *Falls incidence and adverse events*

Falls and adverse events, including fractures, will be ascertained using specific monthly ‘falls calendars’. A fall will be defined as “*unintentionally coming to the ground or some lower level and other than as a consequence of a sudden onset of paralysis, epileptic seizure, or overwhelming external force*” [54]. Participants will be instructed to document any fall and what, if any, medical attention was sought (doctor or hospital visits). At the end of each month participants will return their calendars via reply paid postage envelopes. If a fall is recorded, the participant will be contacted for further details. Any missing calendars will be followed up by research staff. Any injuries, incidents or adverse events that occur during *Osteo-cise* training sessions will be documented by the trainers in an Incident Report Form located in the *Osteo-cise* gym folder.

##### *Anthropometry and body composition*

Height will be measured with a standardised wall mounted stadiometer to the nearest 0.1 cm. Body weight will be measured using digital scales to the nearest 0.1 kg. Total body lean mass, fat mass and percentage body fat will be determined from the total body DXA scan using a Hologic Discovery W dual-energy X-ray absorptiometry (DXA) machine with the APEX Software v3.2 (Hologic, Bedford, MA). All follow-up measurements will be analyzed using the ‘comparison’ feature of the Hologic APEX 3.2 analysis program. The short term co-efficient of variation for repeated measurements of total body lean mass and fat mass in our laboratory is 0.6% and 1.2%, respectively.

#### Other measures

##### *Biochemical and hormonal measures*

Participants will be directed to a local pathology collection centre following each testing visit to provide a fasted, morning (8 to 10 am) blood sample. Blood samples will be tested for serum calcium, albumin, phosphate, creatinine, alkaline phosphatase and C-reactive protein. Remaining serum will be aliquoted and stored at −80°C until completion of the study when samples will be analysed for the following hormonal measures: serum 25-hydroxyvitamin D, parathyroid hormone (PTH) and insulin-like growth factor-1 (IGF-1).

##### *Lifestyle and medical history*

All participants will complete a lifestyle questionnaire detailing education background, current and past employment, history of disease(s)/illnesses, previous fractures, family history of fractures/osteoporosis, current medications and dietary supplements, average weekly alcohol consumption, and weekly television viewing and sitting time. For female participants, menstrual history will be documented including age of onset of menopause, menstrual cycle regularity and history of oral contraceptive and HRT use.

##### *Compliance*

Compliance with the *Osteo-cise* exercise program will be evaluated via self-completed exercise cards that research staff will collect from each health and fitness centre approximately 3-monthly throughout the intervention. Compliance with taking the prescribed calcium and vitamin D supplements will be determined by a medication count when remaining bottles are returned at each testing appointment.

##### *Habitual physical activity*

The CHAMPS physical activity questionnaire will be used to assess current participation in a range of low, moderate, and vigorous physical activities at baseline, 6, 12 and 18 months of the study. This self-report questionnaire has been designed for use in older adults and has been found to be reliable, valid and sensitive to changes in physical activity behaviour [55]. Participants will document their weekly frequency and duration of participation in a ‘typical week’ of the preceding four weeks. The results will be reported as estimated kilojoules per week spent in moderate to high intensity activities. The total number of hours spent performing weight-bearing impact activities will also be determined from this questionnaire.

##### *Diet*

Nutrient intakes will be assessed at each testing visit using 24-hour dietary recall whereby participants will record the type, amount and time of consumption of all food and fluid consumed over a 24-hour period. Participants will be encouraged to provide as much detail as possible including brand names of known foods and exact cooking methods. This questionnaire will be checked for completeness by research staff at each respective testing appointment so that any ambiguous or missing information can be clarified. Dietary information from the questionnaires will be entered and analysed using Australia-specific dietary analysis software (Foodworks, Xyris Software, Highgate Hill, Australia).

##### *Cost Utility Analysis (CUA)*

The CUA will determine the quality of health outcomes averted by the *Osteo-cise: Strong Bones for Life* program. The outcome will be expressed as a cost per quality-adjusted life-years (QALYs) gained. This method of cost analysis involves an assessment of incremental change in health-related quality of life and the monetary costs related to the development and implementation of the *Osteo-cise* program as well as monetary costs related to any adverse events such as injury. The change in quality of life between the groups will be calculated using the difference in QALYs from baseline to the 12-month time point and will be determined using the EuroQoL-5D questionnaire [56].

The EuroQoL-5D considers five dimensions of health, including mobility, self-care, usual activities, pain/discomfort and anxiety/depression. The responses will be scored/weighted using recently published algorithms for Australia [57]. The assessment of monetary costs will include an incremental cost related to program development plus the costs of implementing the program including staff time and costs of all materials/equipment. Costs related to injury or adverse events will also be included (medical care, non-medical care and indirect costs). The Australian Medicare Scheduled fees will be used as uniform unit costs where applicable.

##### *Osteoporosis health beliefs and knowledge*

To evaluate the effect of the program on each participant’s knowledge and health beliefs related to osteoporosis, the Osteoporosis Knowledge Assessment Tool (OKAT) [58] and Osteoporosis Health Belief Scale (OHBS) [59] will be used. The OKT is a 20-item instrument with ‘true’, ‘false’ and ‘don’t know’ response options for measuring osteoporosis knowledge, and has good psychometric properties in Australian adults [58]. Participants are scored on their percentage of correctly answered items. The OHBS questionnaire will measure each participant’s health beliefs related to osteoporosis, exercise behaviours and calcium intake. Participants will rate each of the 42 items on a 5-point scale (1-strongly disagree, 2-disagree, 3-neutral, 4-agree and 5-strongly agree). The OHBS has seven subscales including Seriousness, Health Motivation, Calcium Benefits, Calcium Barriers, Exercise Benefits and Exercise Barriers. The test-retest reliability is 0.90, and 0.71 to 0.82 for the individual subscales of this test [59].

##### *Health-related Quality of Life*

Health-related quality of life will be assessed at each visit using the SF-36 questionnaire, which is a general measure of health status including eight scales: Physical functioning, Role-Physical, Bodily pain, General Health, Vitality, Social Functioning, Role-Emotional and Mental Health [60]. This test yields a score from 0 to 100, where 0 represents the lowest and 100 represents the highest quality of life. Reliability scores of above 0.80 and empirical validity of 0.80 to 0.90 have been reported in the literature for both the physical and mental health measures [60].

### Statistical analyses

Statistical analyses will be computed using STATA statistical software (version 11.0, Stata Corp, College Station, TX). Baseline and demographic data will be presented using descriptive statistics. All intervention data will be analysed according to the principle of intention-to-treat (ITT) and adjusted for multiple comparisons where appropriate. Non- normally distributed data will be transformed prior to analysis. Comparisons between the groups for all continuous primary and secondary outcomes variables will be made using repeated measures analysis of variance (ANOVA) for both time and group-by-time interactions. ANOVA or analysis of covariance (ANCOVA) will be used for absolute and/or percent changes after 12 and 18 months after adjusting for relevant confounders. Falls will be analysed according to the rate of falls per person-year, the proportion sustaining one or more falls and injurious falls during follow-up. The number of falls will be analysed using Poisson regression models with robust standard errors to allow for non-independence of multiple events for the same participant and negative binomial regression models allowing for overdispersion.

Cost utility analysis: Cost data is often skewed and thus not normally distributed. Therefore the Kruskal-Wallis one-way analysis of variance will be used to test the difference in cost and quality of life between the ‘usual care’ and exercise groups. To investigate the difference in cost between the groups, the transformed cost variable will be used as the dependent variable in a multivariable ordinary least square (OLS) regression model. All data will be presented as means ± SD or 95% CI unless stated. The significance level will be *p* < 0.05 or smaller where adjusted for multiple comparisons.

## Discussion

Osteoporosis and associated fractures are a growing public health concern globally, but current practice is largely aimed at secondary prevention after fractures have occurred, rather than primary prevention at the community level [2]. The *Osteo-cise: Strong Bones for Life* project is unique in that it is designed as an evidence-based ‘*research to practise'* trial to evaluate the feasibility and effectiveness of a multifaceted community-based exercise, osteoporosis education/awareness and behavioural change program for improving musculoskeletal health and function in older adults at increased risk of osteoporosis, falls and fractures. Specifically, this project has been designed to: i) provide greater access and increased opportunities for older adults living within the broader community to participate in a safe and evidence-based multifaceted exercise and osteoporosis education program; ii) evaluate the effectiveness of a high velocity PRT in combination with weight-bearing exercise and challenging balance training for improving BMD, body composition, muscle strength and functional performance in older adults at increased risk of falling and fracture; iii) the program incorporates well established behavioural theories and strategies to maximise participant adherence to the program, as well as participant education seminars and written material to maximise osteoporosis-related knowledge and encourage long-term positive changes in health behaviour, and iv) to enhance the capacity for community-based health care professionals and the health and fitness industry to implement an evidence-based targeted osteoporosis and falls prevention program through training and support, and assist them with the establishment of their own sustainable program following the completion of the 12-month intervention. It is intended that the results of the study will inform future clinical practice in order to reduce the global rates of osteoporosis-related fractures.

## Competing interests

The authors declare that they have no competing interests.

## Authors’ contributions

RMD originated the idea for the study and will supervise the project. RMD, PRE, KMS, CAN, KH and CAB were co-investigators of the successful funding proposal. CAB and JG will act as trial coordinators and will be responsible for the data acquisition. JG, CAB and RMD wrote the manuscript and PRE, KMS, CAN and KH reviewed draft versions. All authors have read and approved the final version.

## Pre-publication history

The pre-publication history for this paper can be accessed here:

http://www.biomedcentral.com/1471-2474/13/78/prepub
